# Should All Women with Polycystic Ovary Syndrome Be Screened for Metabolic Parameters?: A Hospital-Based Observational Study

**DOI:** 10.1371/journal.pone.0167036

**Published:** 2016-11-30

**Authors:** Hui Li, Lin Li, Jian Gu, Yu Li, Xiaoli Chen, Dongzi Yang

**Affiliations:** 1 Department of Reproductive Medicine Center, Sun Yat-Sen Memorial Hospital, GuangZhou, China; 2 Department of Gynecology, Third Affiliated Hospital of Sun Yat-Sen University, GuangZhou, China; Zhejiang University College of Life Sciences, CHINA

## Abstract

This hospital-based observational study aims to estimate differences in metabolic abnormalities between different polycystic ovary syndrome (PCOS) phenotypes and their distribution characteristics. The prevalence of metabolic abnormalities among different PCOS phenotypes, including diabetes mellitus (DM), metabolic syndrome (MS), pre-diabetes mellitus (pre-DM), insulin resistance (IR) and dyslipidemia were compared. A total of 2436 women who were ≥18 years old and who were hospitalized in Sun Yat-Sen University affiliated hospital from 1998 to 2015 in GuangZhou, China, were included in this study. PCOS phenotypes were recorded according to the 2003 Rotterdam criteria, including the polycystic ovary morphology (PCO), hyperandrogenism (HA) and ovulation dysfunction (OD) phenotype (PCO+HA+OD); the ovulation phenotype (PCO+HA); the non-PCO phenotype (HA+OD); and the non-HA phenotype (PCO+OD). Notably, 56% of the patients had the classic phenotype (PCO+HA+OD). Importantly, there was no significant difference in the prevalence of metabolic abnormalities or the distribution characteristics of the metabolic abnormalities among these four PCOS phenotypes. Our study supports the notion that metabolic abnormalities and the distribution characteristics of metabolic abnormalities should not be used to distinguish among the various clinical PCOS phenotypes.

## Introduction

Polycystic ovary syndrome (PCOS) is the most common endocrine disorder among women of reproductive age. It has a prevalence of 5.6% in China [1]. PCOS is a complex syndrome with potential effects across the whole life span of affected female patients. Although several clinical treatment options have been suggested, there is an urgent need for a better understanding of this disease to obtain more effective therapeutic strategies. The difficulties arise in large part because of the heterogeneous nature of PCOS [2]. In addition, the collection and evaluation of metabolic abnormality parameters in women with PCOS at their first visit remains controversial, although this information may provide additional and reliable evidence that can be used to guide treatment.

The National Institutes of Health (NIH) in the U.S. recommended in December 2012 that specific PCOS phenotypes should be reported in all research studies and during clinical care [3]. Clinically, the specific phenotypes of PCOS women include a classic phenotype, which presents all three polycystic ovarian morphologies in addition to hyperandrogenism and ovulatory dysfunction (PCO+HA+OD); a phenotype with ovulation in addition to polycystic ovarian morphology and hyperandrogenism (PCO+HA); a phenotype without polycystic ovary morphology but with androgen and ovulatory dysfunction (HA+OD); and a phenotype without hyperandrogenism but with polycystic ovarian morphology and ovulatory dysfunction (PCO+OD). However, these four phenotypes focus only on the reproductive features of PCOS without addressing associated metabolic and cardiovascular disorders [3]. Metabolic abnormality is one of the most severe and long-term complications in PCOS patients, and treatment usually requires a significant amount of effort by the physician which therefore increases the expense for the patient. Hence, if we can predict whether metabolic abnormality is likely to develop when diagnosing PCOS during the reproductive-age years of affected patients, we might be able to provide preventive treatments to minimize the risk of metabolic abnormality and thereby improve the prognosis and maximize the cost-effectiveness of treatments.

However, controversial results have been reported in the years since the NIH made its recommendation [3]. Specifically, Huang et al. [4] analyzed the metabolic profiles of 229 Chinese Taiwanese PCOS patients and suggested that the classic phenotype (PCO+HA+OD) was associated with the most severe metabolic profiles, whereas the PCO+OD phenotype was associated with the least severe profile [4]. However, other studies have argued that the HA phenotype is more closely associated with severe metabolic phenotypes [5–7], and Zhang et al. [8] suggested that there is no difference in the clinical biochemical profiles of patients with different phenotypes. To further address this controversial issue and to analyze the connections between metabolic disorders and PCOS phenotypes, we analyzed the largest PCOS patient database available in southern China. We used first-visit data for patients with PCOS who were diagnosed from 1998 to 2015 to estimate the differences in and the distribution rules of metabolic abnormalities across different PCOS phenotypes. In this study, we provide compelling statistical data supporting the notion that the different PCOS phenotypes are not highly associated with different metabolic abnormalities, and more importantly, we show that PCOS phenotypes cannot be used to reliably predict the development of metabolic abnormality later in life in PCOS patients.

## Materials and Methods

### Participants

A total of 2635 Chinese women who were initially diagnosed with PCOS were included in the PCOS database of Sun Yat-Sen Memorial Hospital (from January 1998 to August 2015). The inclusion criteria were chosen according to the 2003 Rotterdam criteria for PCOS: 1) ovulation dysfunction (OD) or oligo/amenorrhea (less than 8 menstrual cycles per year or more than 35 days per menstrual cycle); 2) hyperandrogenism (HA), clinical hyperandrogenism (hirsutism FG score ≥ 8) and/or biochemical hyperandrogenism (one or more of the following indicators: testosterone (T > 2.6 nmol/L), free testosterone (FT > 4.1 pg/ml), androstenedione (A2 > 3.8 ng/ml), or dihydroepiandrosterone sulfate (DHEA-S > 2170.0 ng/ml) exceeding the upper limits determined by SUN Yat-Sen Memorial laboratory); 3) polycystic ovary morphology (PCO), PCO on ultrasonography (number of follicles ≥ 12 or ovarian volume ≥ 10 cm^3^). PCOS was diagnosed if at least two of these three criteria were present and when other etiologies, such as congenital adrenal hyperplasia, androgen-producing neoplasm, Cushing’s syndrome, thyroid dysfunction, and pituitary prolactinoma, were excluded. According to the inclusion criteria, the patients were divided into one of four reproductive phenotypes [3]: a classic phenotype with polycystic ovarian morphology, hyperandrogenism and ovulatory dysfunction (PCO+HA+OD); a phenotype with ovulation in addition to polycystic ovarian morphology and hyperandrogenism (PCO+HA); a phenotype without polycystic ovary morphology but with androgen and ovulatory dysfunction (HA+OD); and a phenotype without hyperandrogenism but with polycystic ovarian morphology and ovulatory dysfunction (PCO+OD).

### Study design

#### Metabolic parameters and metabolic abnormalities

The glucose diagnostic criteria were based on the definitions of the American Diabetes Association (ADA): 1) the diagnostic criteria for diabetes mellitus (DM) include blood glucose levels higher than 7.0 mmol/L at least 8 hours after fasting or 2-hour glucose levels for OGTT higher than 11.1 mmol/L; 2) the diagnostic criterion for impaired fasting glucose (IFG) was fasting plasma glucose higher than 5.6 mmol/L but less than 7.0 mmol/L; and 3) the diagnostic criterion for impaired glucose tolerance (IGT) was 2-hour plasma glucose OGTT higher than 7.8 mmol/L but less than 11.1 mmol/L. Pre-diabetes mellitus (pre-DM) included IFG and IGT. The diagnostic criteria for insulin resistance (IR) included the fohllowing: 1) HOMA-IR ≥ 2.14 (HOMA-IR = fasting plasma glucose (mmol/L) × fasting insulin levels (mU/mL)); and 2) fasting insulin levels ≥ 12.6 mU/L [9]. IR was diagnosed if both criteria were met. Hypertension was diagnosed if systolic blood pressure (SBP) was higher than 140 mmHg and/or diastolic blood pressure (DBP) was higher than 90 mmHg without taking antihypertensive drugs according to the 1995 criteria of the WHO. Dyslipidemia was diagnosed if one of the following criteria was present: 1) CHOL ≥ 6.0 mmol/L, 2) TG ≥ 1.7 mmol/L, 3) HDL-C < 1.29 mmol/L and 4) LDL-C ≥ 3.6 mmol/L. Metabolic syndrome (MS) was diagnosed if at least two of the following criteria were present in addition to central obesity: waistline ≥ 80 cm [1]. These criteria were determined according to the suggestions of the International Diabetes Federation (IDF): 1) TG ≥ 1.7 mmol/L, 2) HDL < 1.29 mmol/L, 3) SBP ≥ 130 mmHg, DBP ≥ 85 mmHg or previously diagnosed hypertension, and 4) FPG ≥ 5.6 mmol/L or type 2 DM was previously diagnosed.

#### Anthropometric parameters

Waist circumference was measured while the participant was in a standing position. The location of the measurement was halfway between the lower ribs and the superior anterior iliac spine of the pelvis. Hip circumference was measured at the level of the pubic symphysis (WHR = waistline (cm) / hips (cm)). Blood pressure was obtained while the patient was in a sitting position after a five-minute rest. Hirsutism was established using modified Ferriman-Galwey scores [10]. Height and body weight were measured, and BMI values were calculated as follows: BMI = weight (kg) / height (m^2^). The diagnostic standard for obesity was BMI ≥ 23 kg/m^2^ [11]. The diagnostic standard for central obesity was a waist circumference ≥ 80 cm [12].

#### Assays

Prolactin (PRL), luteinizing hormone (LH), follicle stimulating hormone (FSH), estrogen (E2) and testosterone (T) levels were assessed using chemiluminescence (Beckman, USA). FT, sex hormone binding protein (SHBG), DHEAS and 17-OHP levels were assessed using ELISA, and TSH and insulin levels were assessed using chemiluminescence (CPC, USA). Lipids were measured using an automatic biochemical analyzer (Hitachi, Japan). Plasma glucose levels were assessed by analyzing glucose oxidation.

### Statistics

SPSS version 17 was used for the data analyses. Baseline characteristics were presented as median values (upper and lower quartiles) for continuous variables because a normality test for the quantitative data resulted in skewed distributions. Differences among the PCOS phenotypes were detected using nonparametric Wilcoxon signed rank tests. Rates and proportions were calculated for categorical data, and differences were detected using chi-square tests. A *p* value of < 0.05 was considered to indicate statistical significance. Hypothesis testing was performed at a level of **α** = 0.05.

### Ethics

This study was a hospital-based retrospective study. All data were obtained from the 1PCOS database of Sun Yat-Sen Memorial Hospital. This study is one component of the hospital 5010 clinical research program, which is a long-term follow-up study of metabolic abnormality and clinical outcomes in patients with PCOS at Sun Yat-Sen University. This project was approved by the ethics committee of Sun Yat-Sen Memorial Hospital. All patients signed an informed consent document consenting to the scientific use of their data.

## Results

### PCOS phenotypes

**Fig 1:**
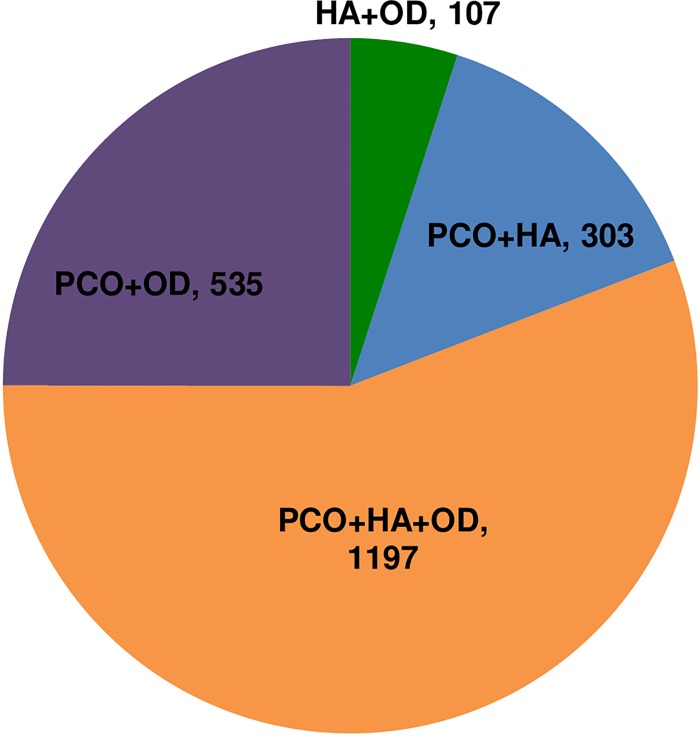
Distribution of PCOS Phenotypes in Female Participants (≥ 18 years of age). [PCO+HA+OD is the classic reproductive phenotype which has polycystic ovary morphology, androgen excess and ovulation dysfunction. PCO+HA is the ovulatory phenotype which has polycystic ovary morphology and androgen excess with ovulation. HA+OD is the phenotype which has androgen excess and ovulation dysfunction without polycystic ovary morphology. PCO+OD is the phenotype which has polycystic ovary morphology and ovulation dysfunction without androgen excess. Among 2436 young adults with PCOS (≥ 18 years of age), (1197/1981) were PCO+HA+OD, (303/2215) were PCO+HA, (107/2116) were HA+OD, and (535/1997) were PCO+OD.]

According to the 2003 Rotterdam criteria, each PCOS patient was characterized as having one of four phenotypes [3]. The phenotypes were based on reproductive features and included a classical phenotype (PCO+HA+OD), which presented polycystic ovary morphology, hyperandrogenism and ovulation dysfunction; an ovulation phenotype (PCO+HA) that presented polycystic ovary morphology and hyperandrogenism, a phenotype with hyperandrogenism and ovulation dysfunction (HA+OD), and a phenotype with polycystic ovary morphology and ovulation dysfunction (PCO+OD). In our study, the proportion of patients with PCO was 2172/2237 (positive value/valid value) (97.1%), the proportion of patients with HA was 1644/2388 (68.8%), and the proportion of patients with OD was 1904/2142 (88.9%). Overall, 1197 (56%) patients were diagnosed with the classical phenotype (PCO+HA+OD), 303 patients (14%) were diagnosed with the ovulation phenotype (PCO+HA), 107 patients (5%) were diagnosed with the HA+OD phenotype, and 535 patients (25%) were diagnosed with the PCO+OD phenotype. Among the four phenotypes, it was clear that the classical phenotype (PCO+HA+OD) represented the largest proportion of patients at diagnosis (first visit) (Fig 1).

### Parameters of participants

A total of 2635 female patients were diagnosed with PCOS in the outpatient clinic of the department of Obstetrics and Gynecology of Sun Yat-Sen Memorial Hospital from January 1998 to August 2015. Among these patients, 2436 were ≥ 18 years of age, and the median age was 27.0 years. Significant differences were observed in age between the PCOS phenotypes (*p* = 0.000): the phenotype without hyperandrogenism (PCO+OD) was observed in most elderly patients, whereas the phenotype without PCO (HA+OD) was more frequently observed in the youngest patients (Table 1). Among all participants, the median waist circumference was 75.5 cm, the median BMI was 21.56 kg/m^2^, and the median WHR was 0.83. There were no significant differences in WHR or BMI among the four PCOS phenotypes (Table 1).

**Table 1:** Anthropometric, plasma glucose and lipid parameters of participants.

Parameter(Median(upper, lower quartiles))	HA+OD(N = 107)	PCO+HA(N = 303)	PCO+HA+OD(N = 1197)	PCO+OD(N = 535)	P value
Age, year	25.00(21.00,29.00)	27.00(23.00,30.00)	27.00(23.00,29.00)	28.00(25.00,30.00)	**0.000**
**BP, Median(upper, lower quartiles)**					
Systolic BP, mmHg	113.00(104.00,122.00)	112.50(104.00,124.75)	114.00(105.00,123.00)	114.00(106.00,122.00)	0.930
Diastolic BP, mmHg	74.00(66.00,81.00)	74.00(70.00,80.00)	74.00(68.00,80.00)	75.00(69.25,80.00)	0.385
**Anthropometrics, Median(upper, lower quartiles)**					
Waist circumference, cm	75.00(70.00,83.50)	75.00(68.25,83.50)	76.00(70.00,84.00)	75.00(70.00,83.00)	0.766
WHR	0.82(0.78,0.87)	0.82(0.77,0.87)	0.83(0.78,0.88)	0.83(0.78,0.87)	0.260
Height, cm	159.00(155.00,161.50)	159.00(156.00,163.00)	158.00(155.00,162.00)	158.00(155.00,161.00)	0.117
Weight, kg	54.00(49.00,60.00)	55.00(49.00,62.05)	55.00(49.00,62.00)	53.00(49.00,60.00)	0.231
BMI, kg/m2	21.10(19.45,24.10)	21.63(19.14,24.45)	21.83(19.47,25.00)	21.23(19.38,23.96)	0.239
**OGTT**					
FPG, mmol/l	5.00(4.70,5.30)	5.10(4.70,5.40)	5.00(4.70,5.30)	5.00(4.70,5.30)	0.336
1hour PG, mmol/l	7.75(6.00,10.05)	8.10(6.40,9.80)	8.10(6.52,9.70)	7.70(6.30,9.40)	0.060
2hour PG, mmol/l	6.20(5.30,7.50)	6.40(5.30,7.58)	6.30(5.40,7.70)	6.30(5.30,7.48)	0.775
HbA1C, %	5.20(4.90, 5.40)	5.20(4.90,5.40)	5.20(5.00,5.50)	5.20(4.90,5.50)	0.866
**Insulin**					
Fins, mU/L	10.60(5.83, 17.10)	8.25(5.54,13.59)	9.55(5.83,15.19)	8.75(5.14,14.18)	**0.041**
1hour Ins, mU/L	92.05(61.61,135.50)	83.75(52.00,131.00)	91.10(58.30,161.95)	82.15(50.25,146.00)	**0.004**
2hour Ins, mU/L	73.90(48.70,146.00)	65.69(43.62,120.00)	73.06(46.07,142.00)	61.35(39.33,120.92)	**0.005**
**Lipid**					
CHOL, mmol/L	4.66(4.34,5.27)	4.86(4.32,5.50)	4.86(4.31,5.47)	4.79(4.23,5.44)	0.274
TG, mmol/L	1.09(0.88,1.51)	1.09(0.77,1.53)	1.08(0.76,1.62)	1.03(0.76,1.54)	0.716
HDL, mmol/L	1.43(1.28,1.71)	1.47(1.21,1.78)	1.46(1.25,1.74)	1.48(1.26,1.72)	0.982
LDL, mmol/L	2.82(2.27,3.29)	2.97(2.41,3.49)	2.91(2.44,3.43)	2.79(2.37,3.34)	0.199
APOA, g/L	1.25(1.15,1.46)	1.30(1.17,1.49)	1.31(1.14,1.52)	1.29(1.14,1.50)	0.894
APOB, g/L	0.68(0.60, 0.77)	0.73(0.62,0.86)	0.75(0.65, 0.88)	0.75(0.63, 0.86)	**0.010**

APOA: apolipoprotein A, APOB: apolipoprotein B, BMI: body mass index, CHOL: cholesterol, FIN: fasting insulin level, HA+OD is the phenotype which has androgen excess and ovulation dysfunction without polycystic ovary morphology, HDL: high-density lipoprotein, LDL: low-density lipoprotein, 1hour Ins: insulin level on 1hour, 2hour Ins: insulin level on 2 hour. FPG: fasting plasma glucose, 1hour PG: plasma glucose on 1hour, 2hour PG: plasma glucose on 2 hour. PCO+HA+OD is the classic reproductive phenotype which has polycystic ovary morphology, androgen excess and ovulation dysfunction. PCO+HA is the ovulatory phenotype which has polycystic ovary morphology and androgen excess with ovulation. PCO+OD is the phenotype which has polycystic ovary morphology and ovulation dysfunction without androgen excess. TG: triglycerides, WHR: waist to hip ratio.

### Distribution of metabolic abnormality in PCOS phenotypes

**Fig 2:**
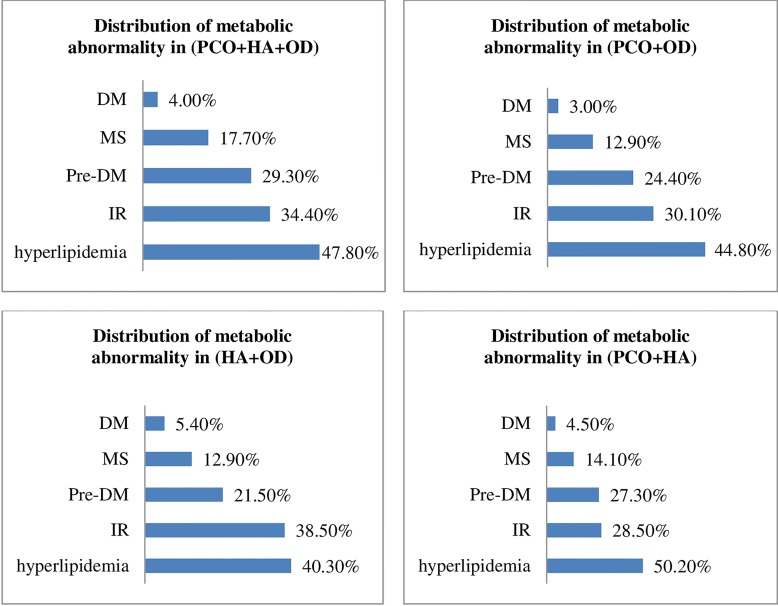
Distribution of metabolic abnormalities in the indicated PCOS phenotypes. [DM: diabetes mellitus, IFG: impaired fasting glucose, IGT: impaired glucose tolerance, IR: insulin resistance, MS: metabolic syndrome, pre-DM: pre-diabetes mellitus, including IFG and IGT. PCO+HA+OD is the classic reproductive phenotype which has polycystic ovary morphology, androgen excess and ovulation dysfunction. PCO+HA is the ovulatory phenotype which has polycystic ovary morphology and androgen excess with ovulation. PCO+OD is the phenotype that has polycystic ovary morphology and ovulation dysfunction without androgen excess. HA+OD is the phenotype that has androgen excess and ovulation dysfunction without polycystic ovary morphology.]

We analyzed the relationships between the recorded metabolic disorders and the PCOS phenotypes to determine if we could predict whether metabolic abnormality would develop at a later stage according to the PCOS phenotypes diagnosed earlier. No significant changes were observed in the distribution characteristics associated with metabolic abnormality in any of the four PCOS phenotypes (Fig 2). For example, DM had the lowest frequency in all phenotypes, accounting for 4.00% of the classical phenotype (PCO+HA+OD), 3.00% of the PCO+OD phenotype, 5.40% of the HA+OD phenotype and 4.50% of the PCO+HA phenotype. Conversely, dyslipidemia was the most frequently observed metabolic abnormality in all four phenotypes, and it was detected in nearly half of all patients (Fig 2). IR, pre-DM and MS were also observed in patients and ranged between 10% and 40% (Fig 2).

Consistent with previous reports, various types of metabolic abnormalities were observed in a significant proportion of the female PCOS patients. The observed metabolic abnormalities included obesity in 765/2110 (positive value/valid value) (36.3%) patients; central obesity in 699/1879 (37.2%) patients; IR in 639/1952 (32.7%) patients; IFG in 267/1979 (13.5%) patients; IGT in 372/1877 (19.8%) patients; pre-DM in 545/1982 (27.5%) patients; DM in 78/1982 (3.9%) patients; hypertension in 342/1716 (19.9%) patients; dyslipidemia in 763/1621 (47.1%) patients and MS in 270/1705 (15.8%) patients.

Importantly, we observed statistical differences in the plasma insulin and APO-B (apolipoprotein-B) parameters (*p* < 0.05) (Table 1) but not in the prevalence of all metabolic abnormalities among the four PCOS phenotypes (Table 2). These results suggest, in part, that PCOS phenotypes may predict plasma insulin and APO-B abnormalities but not metabolic abnormalities.

**Table 2:** Prevalence of Metabolic Disorders Among the PCOS Phenotypes (age ≥ 18).

Parameter(No./valid value (%))	HA+OD(N = 107)	PCO+HA(N = 303)	PCO+HA+OD(N = 1197)	PCO+OD(N = 535)	P value
**Glucose metabolic disorders**					
DM	5/93(5.4%)	13/286(4.5%)	46/1136(4.0%)	14/467(3.0%)	0.590
IFG	9/93(9.7%)	46/285(16.1%)	160/1135(14.1%)	52/466(11.2%)	0.145
IGT	14/81(17.3%)	55/268(20.5%)	223/1084(20.6%)	80/444(18.0%)	0.636
Pre-DM	20/93(21.5%)	78/286(27.3%)	333/1136(29.3%)	114/467(24.4%)	0.122
Obesity	36/105(34.3%)	111/297(37.4%)	451/1182(38.2%)	167/526(31.7%)	0.078
Central obesity	29/85(34.1%)	91/249(36.5%)	416/1070(38.9%)	163/475(34.3%)	0.338
IR	35/91(38.5%)	80/281(28.5%)	386/1122(34.4%)	138/458(30.1%)	0.089
**Other metabolic disorders**					
MS	9/70(12.9%)	32/227 (14.1%)	176/997 (17.7%)	53/411(12.9%)	0.106
Hyperlipidemia	27/67(40.3%)	107/213 (50.2%)	454/950 (47.8%)	175/391(44.8%)	0.373

DM: diabetes mellitus, IFG: impaired fasting glucose, IGT: impaired glucose tolerance, IR: insulin resistance, MS: metabolic syndrome, pre-DM: pre-diabetes mellitus, including IFG and IGT. HA+OD is the phenotype which has androgen excess and ovulation dysfunction without polycystic ovary morphology,. PCO+HA+OD is the classic reproductive phenotype which has polycystic ovary morphology, androgen excess and ovulation dysfunction. PCO+HA is the ovulatory phenotype which has polycystic ovary morphology and androgen excess with ovulation. PCO+OD is the phenotype which has polycystic ovary morphology and ovulation dysfunction without androgen excess.

After normalizing for age and BMI, the risk of IFG in patients with the phenotype without hyperandrogenism (PCO+OD) was 0.699 (*p* = 0.043), while the risk of pre-DM was 0.719 (*p* = 0.014) and MS was 0.562 (*p* = 0.005). In addition, the risk of MS in the ovulation phenotype (PCO+HA) was 0.611 (*p* = 0.047) (Table 2). However, there were no significant differences in glucose or lipid metabolic abnormalities between the patient groups with the HA+OD phenotype and those with the PCO+HA+OD phenotype even when the results were adjusted for age and BMI. Collectively, these data indicate that the reason that PCOS phenotypes are not highly associated with metabolic abnormality may be because of the influences of age, height and body weight.

## Discussion

### Distribution of PCOS phenotypes

Among the four PCOS phenotypes, which were defined according to NIH guidelines [3], the classical phenotype (PCO+HA+OD) was the most common phenotype at the first visit in our hospital-based retrospective study. This result is consistent with the results of a Korean study [13] and another clinical study that was performed in China [7]. However, these four PCOS phenotypes were observed to be evenly distributed in one large community-based study [1]. In addition to sample size, most of the studies that have been performed around the world have argued that there are also racial differences in the distribution patterns of PCOS phenotypes [14–17]. However, this was not supported by a case-control study that was performed by Ladson et al. [18], which revealed that differences between black and white PCOS women were minimal.

### Distribution of metabolic abnormalities in PCOS phenotypes

Our observational study and statistical analyses demonstrate that the different metabolic abnormalities were similarly distributed in each of the four first-visit PCOS phenotypes [3]. We found no significant differences in metabolic abnormalities among the PCOS phenotypes. Specifically, the prevalence of metabolic abnormalities in each phenotype, from lower to higher, was DM, MS, pre-DM, IR, and dyslipidemia. Among pre-DM patients, the prevalence of IFG was lower than the prevalence of IGT in all PCOS phenotypes. This observation is in agreement with the results of a previous Korean observational study that included more than 800 PCOS patients and 13 medical locations [13] and that examined the distribution of metabolic abnormalities across various phenotypes. Moreover, our observations also echo those of a large Chinese community-based study [1] in which no significant differences were observed in the prevalence of metabolic abnormalities among PCOS phenotypes. However, in contrast to our study, the reported prevalences of different types of metabolic abnormalities in each phenotype were, from lower to higher, IR, MS and dyslipidemia, and the study lacked data on DM and pre-DM. Discrepancies in metabolic abnormalities among PCOS phenotypes have been noted previously. For example, Huang et al. [4] argued that the PCOS phenotypes represent different severities of metabolic abnormality, with the classical phenotype (PCO+HA+OD) having the most severe metabolic profile. Finally, another study from Zhang et al. [8] showed that there was no statistical difference in the prevalence of different metabolic abnormalities among PCOS phenotypes.

Age and BMI have been indicated to be key factors that may influence metabolic normality. In a national diabetes study [19] that covered 14 provinces and cities, in females, the prevalence of DM was 9.7%, and the prevalence of pre-DM was 15.5%. Notably, that study [19] revealed that the incidence of DM increased with age and BMI, and the prevalence of DM was 3.2% in patients from 20 to 39 years of age. All of the participants in our study were 18 to 44 years of age. The observed prevalence of DM in PCOS was 3.9%, which is consistent with a previous diabetes study [19]. In our study, the proportion of patients with pre-DM was 27.5%, which is higher than the proportion reported in the diabetes study [19], which included a larger population. The prevalence of both DM and pre-DM was higher in the PCOS population in our study than in the national diabetes study [19].

Metabolic abnormalities were distributed similarly among each of the four first-visit PCOS phenotypes, but some differences in metabolic parameters, serum insulin levels and APOB remained among the four phenotypes before adjusting for age. After adjusting for age, there were no significant differences in serum insulin levels (p > 0.05, FIN p = 0.347, 1 hour Ins p = 0.232, 2 hour Ins p = 0.883), but APOB was significantly different among the four phenotypes (p = 0.001). The phenotype (PCO + HA) had the highest level of APOB. OD was a protective factor, and APOB was 1.064 g/l lower in the women with PCOS with OD than in those without OD (p = 0.025). APOB was higher in the women with PCOS with HA or PCO than in those without HA or PCO, but this difference was not statistically significant (p > 0.05). A study of adolescent PCOS in our hospital confirmed that APOB/APOA1 is a good predictor of MS and that the ratio was higher in women with PCOS with obesity and a high free androgen index [20]. Although the clinical study of adolescent PCOS in our hospital did not include clinical manifestations [20], this study was the first to report that OD is a protective factor of APOB. APOB is an easily oxidized substance that induces an inflammatory reaction and the formation of plaques in the arterial wall. Therefore, APOB is probably a high-risk factor for CVD, but the exact value of APOB in the prevalence of CVD remains unknown [21]. We need a long-time follow up study to balance the conflict between maintaining regular menses to prevent endometrial carcinoma and reducing APOB with OD to prevent CVD.

### Strengths and limitations

The distinct strength of our study is that our database is currently the largest PCOS case library in southern China. A huge amount of effort has been expended to produce this powerful dataset over the last 17 years, and it has been very helpful for PCOS research. A limitation of our study is that the included patients are in part biased because most of the included female patients are infertile or have menstrual disorders. In addition, the study was a single hospital-based retrospective study and therefore suffers from location-related limitations. Nonetheless, the relatively large number of patients (more than 2000) who were involved in the study is expected to reduce the bias to some extent. Moreover, although the 2003 Rotterdam diagnostic criteria have been accepted around the world, none of its three grouping criteria are very specific [22], and there are differences related to ethnicity [15]. For example, the standard for OD was a menstrual cycle longer than 35 days, but a regular period probably includes ovulatory dysfunction. HA was diagnosed when an androgen parameter exceeded the limit of the local laboratory or when hirsutism was presented, but the methods used to test androgens vary greatly around the world, and there is currently no gold standard. LC-MS measurements [23,24] were recently proposed as a reliable measure for androgens, but the high cost and lack of clinical feasibility significantly hinder the clinical application of LC-MS. In addition, the phenotype classification method described in the NIH recommendations was also based on the 2003 Rotterdam criteria [3]. However, the 2003 Rotterdam criteria have been a subject of debate for approximately ten years. In 2006, the hyperandrogenism and PCOS society (AE-PCOS) recommended another set of criteria that includes HA [25].

## Conclusions

The data in our study show that the classical phenotype (PCO+HA+OD) is the most common PCOS phenotype that is diagnosed at the first visit in female patients between 18 and 44 years of age in southern China. In this hospital-based observational study, there was no significant difference in the prevalence of each examined metabolic abnormality among the PCOS phenotypes. In conclusion, although our data indicate that PCOS phenotypes cannot be used to predict metabolic disorders, we suggest that all patients with PCOS should be fully evaluated to determine baseline metabolic parameters on their first visit to maximize the potential for preventing the development of metabolic abnormality.
