# Correction: Should All Women with Polycystic Ovary Syndrome Be Screened for Metabolic Parameters?: A Hospital-Based Observational Study

**DOI:** 10.1371/journal.pone.0176806

**Published:** 2017-04-26

**Authors:** Hui Li, Lin Li, Jian Gu, Yu Li, Xiaoli Chen, Dongzi Yang

There are errors in the Funding section. The correct funding information is as follows: This study was supported by Sun Yat-Sen University (no. 2014005, http://research.sysu.edu.cn), the Natural Science Foundation of China (no. 8140060623, http://www.nsfc.gov.cn), the Science Technology Research Project of Guangzhou City (no. 2014Y2-00512, http://wsbs.gzsi.gov.cn), and the Natural Science Foundation of Key Research Project of Guangdong Province (no. 2013020012660, http://pro.gdstc.gov.cn/egrantweb/).
